# Grafting the Tomato on the Potato

**Published:** 1881-06

**Authors:** 


### GRAFTING THE TOMATO ON THE POTATO.

Mr. Hiram Stidolph, of Jefferson County, Ala., writing to the *Rural World*, in October, says:

"I have this summer grafted a tomato vine on a potato vine. It is now growing finely. If it had not been such a dry summer I think it would now be full of fruit, and I should probably have potatoes at one end of the vine and tomatoes at the other end. It has been grafted four months and is now (October 15) full of blossoms. It is the most singular piece of grafting I have ever done. I have grafted white currants upon black currants and on red ones, and a gooseberry on a currant, which bore a gooseberry the first year. But grafting the tomato on the potato gave me more trouble than any grafting I ever did. The tomato vine looked sickly for a long time, but I shaded and watered it, and it finally grew and produced blossoms, as I have stated."

Another correspondent in the same paper, of November 25, adds: "In 1867 an Alsatian gardener, then in my employ, mentioned to me this rather curious fact of the practicability of grafting the tomato on the potato. I think I have since seen it referred to in several works. The tomato may be successfully grafted on the potato. By this means one gets a crop of potatoes in the ground and a crop of tomatoes on the stems. The potatoes (*S. tuberosum*) and the tomato (*S. lycopersicum*) being both Solanaceæ, the inference is that they can be united by proper care in manipulation, and subsequent protection of the graft. The great difficulty, however, in grafting herbaceous plants will prevent its being practiced even by those who have a penchant for oddities.

While nothing useful is gained by the experiment, yet it shows what wonderful transformations can be made in the vegetable kingdom.

